# Clinicopathological impacts of high c-Met expression in renal cell carcinoma: a meta-analysis and review

**DOI:** 10.18632/oncotarget.20796

**Published:** 2017-09-08

**Authors:** Jung Han Kim, Bum Jun Kim, Hyeong Su Kim

**Affiliations:** ^1^ Division of Hemato-Oncology, Department of Internal Medicine, Kangnam Sacred-Heart Hospital, Hallym University Medical Center, Hallym University College of Medicine, Seoul 07441, Republic of Korea; ^2^ Department of Internal Medicine, National Army Capital Hospital, The Armed Forces Medical Command, Sungnam 13574, Republic of Korea

**Keywords:** c-Met, renal cell carcinoma, prognosis, meta-analysis

## Abstract

c-Met overexpression has been observed in renal cell carcinoma (RCC). However, its clinicopathological impacts remain uncertain. We performed this meta-analysis to evaluate the pathologic and prognostic impacts of high c-Met expression in patients with RCC. A systematic computerized search of the electronic databases PubMed and Embase was performed. From 12 studies, 1,724 patients with RCC were included in the meta-analysis. Compared with RCCs showing low c-Met expression, tumors with high c-Met expression showed significantly higher nuclear grade (odds ratio = 2.45 [95% CI: 1.43–4.19], *P* = 0.001) and pT stage (odds ratio = 2.18 [95% CI: 1.27–3.72], *P* = 0.005). In addition, patients with c-Met-high RCC showed significantly worse overall survival than those with c-Met-low tumor (hazard ratio = 1.32 [95% CI: 1.12–1.56], *P* = 0.0009). In conclusion, this meta-analysis demonstrates that high c-Met expression correlate with significantly worse pathological features and overall survival, indicating c-Met overexpression is a potential adverse prognostic marker for patients with RCC.

## INTRODUCTION

Renal cell carcinoma (RCC) is the most common malignant renal neoplasm, accounting for approximately 85% of kidney cancers [1, 2]. Most patients without metastases can be cured by nephrectomy alone. However, considerable patients have metastatic diseases at the time of diagnosis and nephrectomy is not usually curative for those patients [2, 3]. Until the last decade, immunotherapeutic agents (interferon-α and interleukin-2) had been the main treatment option for patients with metastatic RCC, despite marginal benefits and significant toxicities [4, 5].

With understanding of molecular mechanisms of carcinogenesis, treatment of RCC has dramatically changed. The molecular targeted agents such as sorafenib, sunitinib, axitinib, pazopanib, or temsirolimus are currently recommended with improved outcomes for patients with metastatic RCC [6–10]. However, most tumors eventually develop resistance and their survival benefits are still disappointing. Therefore, efforts to identify novel therapeutic targets and develop more effective targeted drugs are still required. c-Met has recently emerged as a possible therapeutic target in various tumors including RCC [11–13].

c-Met is the tyrosine kinase receptor for hepatocyte growth factor (HGF) and encoded by the proto-oncogene *MET* located on chromosome 7. The HGF-c-Met signaling pathway regulates multiple cellular functions, including differentiation, proliferation, and angiogenesis [14, 15]. Thus, dysregulation of c-Met and HGF has been implicated in the pathogenesis of cancers. It is related to molecular mechanisms of tumor cell proliferation and survival, invasion, and metastasis [16]. The enhanced expression of c-Met has been observed in various tumors, such as breast cancer [17], lung cancer [18], gastric cancer [19], colorectal cancer [20], cervix cancer [21], pancreatic cancer [22], and hepatocellular carcinoma [23]. Several meta-analyses in common tumors indicated that high c-Met expression was associated with a poor prognosis [17–23].

The expression of c-Met has also been observed in various cytomorphologic subtypes of RCC [24–39]. High c-Met expression has been associated with poor pathologic features and prognosis in many studies [24–26, 28–31]. However, most studies had a small number of patients, and there has been some conflicts regarding its clinicopathological impacts in RCC [27, 32–34,38, 39]. Therefore, we performed this meta-analysis to evaluate the pathologic and prognostic roles of c-Met overexpression in patients with RCC.

## RESULTS

### Results of search

**Figure 1:**
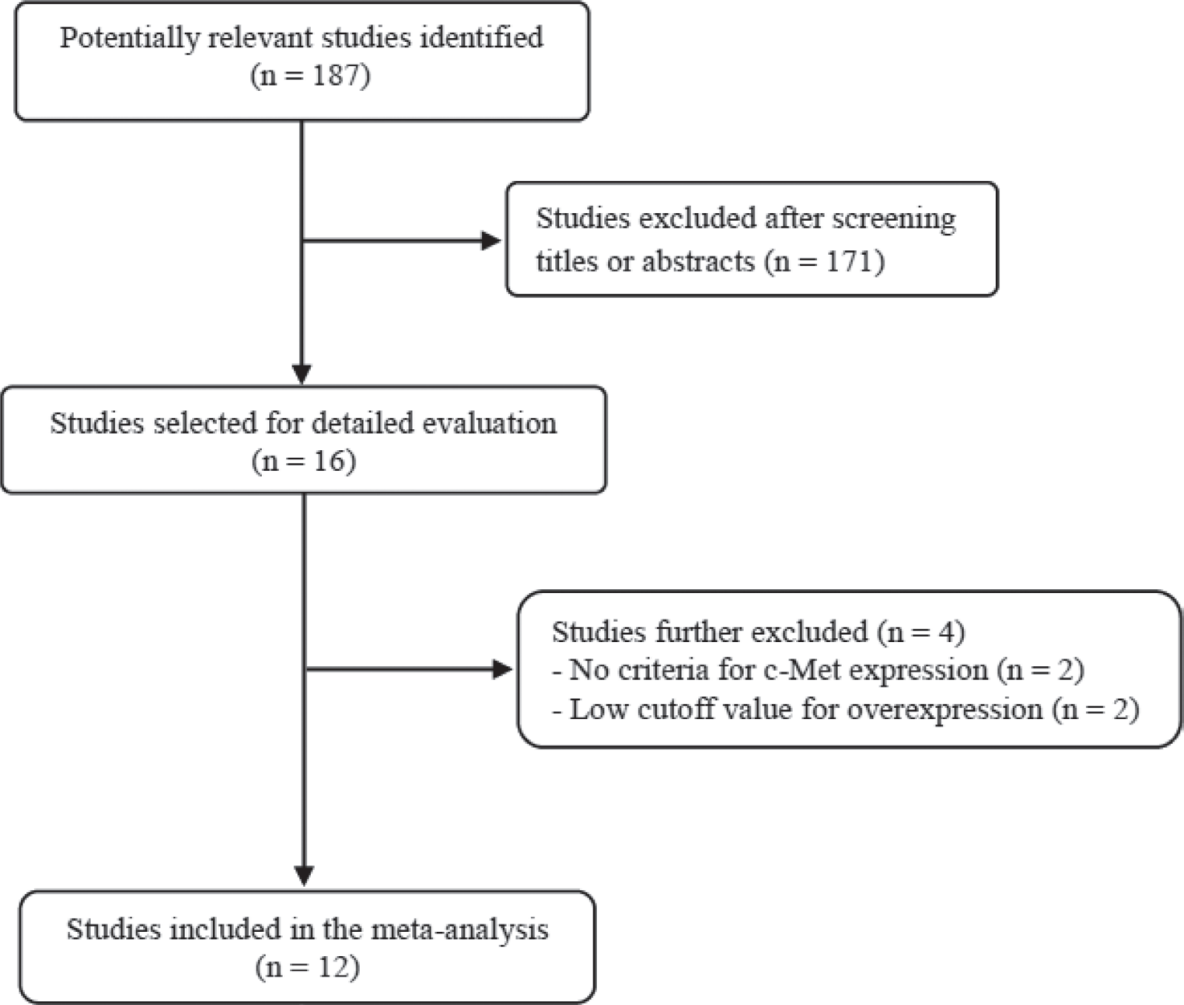
Flow diagram of search process

Figure 1 shows flow diagram of search process. A total of 187 relevant studies were initially retrieved, but 171 of them were excluded after screening the titles and abstracts. Of the remaining 16 potentially eligible studies, 4 were further excluded by the inclusion criteria: two had no criteria for c-Met expression status [24, 25] and the others adopted too low cutoff values (< 10%) for high c-Met expression [26, 27]. Finally, 12 studies were included in the meta-analysis [28–39].

### Characteristics of the included studies

Table 1 summarizes the main characteristics and clinicopathological outcomes of the 12 included studies. All the studies were performed retrospectively. From the 12 studies, 1,724 patients were included in the meta-analysis. In one study, patients (*n* = 81) had chromophobe RCC [35]. Two small studies had patients (*n* = 96) only with papillary RCC (pRCC) [30, 33] and other three had patients (*n* = 752) only with clear cell RCC (ccRCC) [36, 38, 39]. Almost all patients had received renal surgery as primary treatment for RCC. In two studies [38, 39], patients were treated with sunitinib as a first-line therapy for metastatic RCC. Except for one [32], eleven studies used immunohistochemistry (IHC) to assess c-Met expression status [28–31, 33–39].

**Table 1:** Summary of the 12 included studies

Author (year) Country	Histology	Methods, antibody, detection kit or immunostainer	No. of pts	Criteria for c-Met^high^	c-Met^high^ (%)	OR for NG (95% CI)	OR for pT stage (95% CI)	HR for OS (95% CI)
Pisters *et al.*, (1997) USA	RCC	IHC with whole slides, Rabbit c-Met Ab	41	≥ 30% of cancer cells	28 (68.3%)	8.1 (0.75–87.23)	0.99 (0.26–3.70)	NA
Inoue *et al.*, (1998) Japan	RCC	IHC with whole slides, c-Met c-12	120	Positive: higher membrane staining than normal kidney (c-Met^high^: ≥ 50% cancer cells with positivity)	20 (16.7%)	2.97 (1.06–8.36)	0.84 (0.25–2.75)	NA
Sweeney *et al.*, (2002) USA	pRCC	IHC with whole slides, NA	50	Intensity: 0 (absent) to 3 ( intense) (c-Met^high^: ≥ positive cytoplasmic or membrane staining grade 1 or higher in ≥ 10% of cancer cells)	40 (80%)	1.83 (0.45–7.51) *P* = 0.157	12.76 (0.7 – 233.48) *P* = 0.004	6.93 (0.92–52.23) *P* = 0.07
Miyata *et al.*, (2006) Japan	RCC	IHC with whole slides, Phosphorylated c-Met Ab, DAKO EnVision	114	> 50% of cancer cells with higher staining than normal kidney	73 (64%)	1.70 (0.77–3.75)	2.52 (0.93–6.64)	2.94 (1.12–7.72) *P* = 0.028
Betsunoh *et al.*, (2007) Japan	RCC	RT-PCR	66	Tumor/normal ratio ≥ 3	17 (25.8%)	NA	NA	3.16 (0.10 – 92.3) *P* = 0.505
Gontero *et al.*, (2008) Italy	pRCC	IHC with whole slides, Clone DQ 13	46	Cytoplasmic staining ≥ 30% of cancer cells	13 (28.3%)	NA	NA	0.96 (0.38–2.44) *P* = 0.609
Gibney *et al.*, (2012) USA	RCC	IHC with TMA, Anti-c-Met Ab (MET4)	317	≥ Cutoff point of median AQUA score (32.5%)	159 (50.2%)	NA	NA	1.36 (1.08–1.74) *P* = 0.0091
Erlmeier *et al.*, (2013) Germany	chRCC	IHC with TMA, Anti-MET (AB-103), EnVision Kit	81	Intensity: 0 = no staining; 1 = weak; 2 = moderate; 3 = strong Proportion of staining area: recorded in percent (0–100%) (c-Met^high^: intensity score × percentage score > median)	24 (29.6%)	NA	1.89 (0.39–9.19) *P* = 0.6	1.90 (0.42–8.57) *P* = 0.59
Chen *et al.*, (2017) China	ccRCC	IHC with TMA Rabbit anti-c-Met Ab, SP-9000 SP link Kit	90	Intensity: 0 = no signal; 1 = weak; 2 = moderate; 3 = strong Positive rate: 0 = no; 1 = 1–25%; 2 = 26–50%; 3 = 51–75%; 4 = 76–100% (c-Met^high^: intensity score × positive rate score ≥ 6)	15 (16.7%)	10.42 (1.30–83.37)	7.46 (2.16–25.75)	2.85 (1.21–6.70) *P* = 0.017
Peltola *et al.*, (2017) Finland	RCC	IHC with whole slides, Anti-c-Met ready-to-use Mab, BenchMark XT	137	Intensity: 0 = no staining; 1 = weak; 2 = strong; 3 = very strong (c-Met^high^: intensity score 2 or 3)	59 (43.1%)	NA	NA	1.22 (0.81–1.82) *P* = 0.34
Macher-Goeppinger *et al.*, (2017) Germany	ccRCC	IHC with TMA, Anti-total c-Met (SP44), OptiView DAB IHC Kit	572	Intensity: 0 = negative; 1 = low; 2 = medium; 3 = high Quantity: 0 = no expression; 1 = < 10% of positive cells; 2 = positive in 10–50%; 3 = positive in 51–80%; 4 = positive in ≥ 80% (c-Met^high^: intensity score × quantity score ≥ 6)	184 (32.2%)	NA	NA	1.05 (0.69–1.61) *P* = 0.81
Kammerer-Jacquet et al., (2017) France	ccRCC	IHC with whole slides Anti-total c-Met (SP44), BenchMark XT	90	Intensity: 0 = absent; 1 = weak; 2 = moderate; 3 = strong (c-Met^high^: intensity score 2 or 3)	62 (68.9%)	NA	NA	0.99 (0.56–1.78)

RCC, renal cell carcinoma; ccRCC, clear cell RCC; chRCC, chromophobe RCC; pRCC, papillary RCC; IHC, immunohistochemistry; TMA, tissue microarray; Ab, antibody; Mab, monoclonal antibody; RT-PCR, reverse transcription-polymerase chain reaction; pts, patients; AQUA, automated quantitative analysis; OR, odds ratio; HR, hazard ratio; CI, confidence interval; OS, overall survival; NA, not available

### c-Met expression assignation

There was a marked heterogeneity between the criteria used to dichotomize c-Met status (low c-Met or high c-Met). The criteria were briefly summarized in the Table 1. The rates of high c-Met expression were various, ranging from 16.7% [29, 36] to 80% [30].

### Impact of high c-Met expression on pathological features

From six studies [28–31, 35, 36], 496 patients were included in the meta-analysis of odds ratios (ORs) with 95% confidence intervals (CIs) for nuclear grade and/or depth of cancer penetration (pT stage).

**Figure 2:**
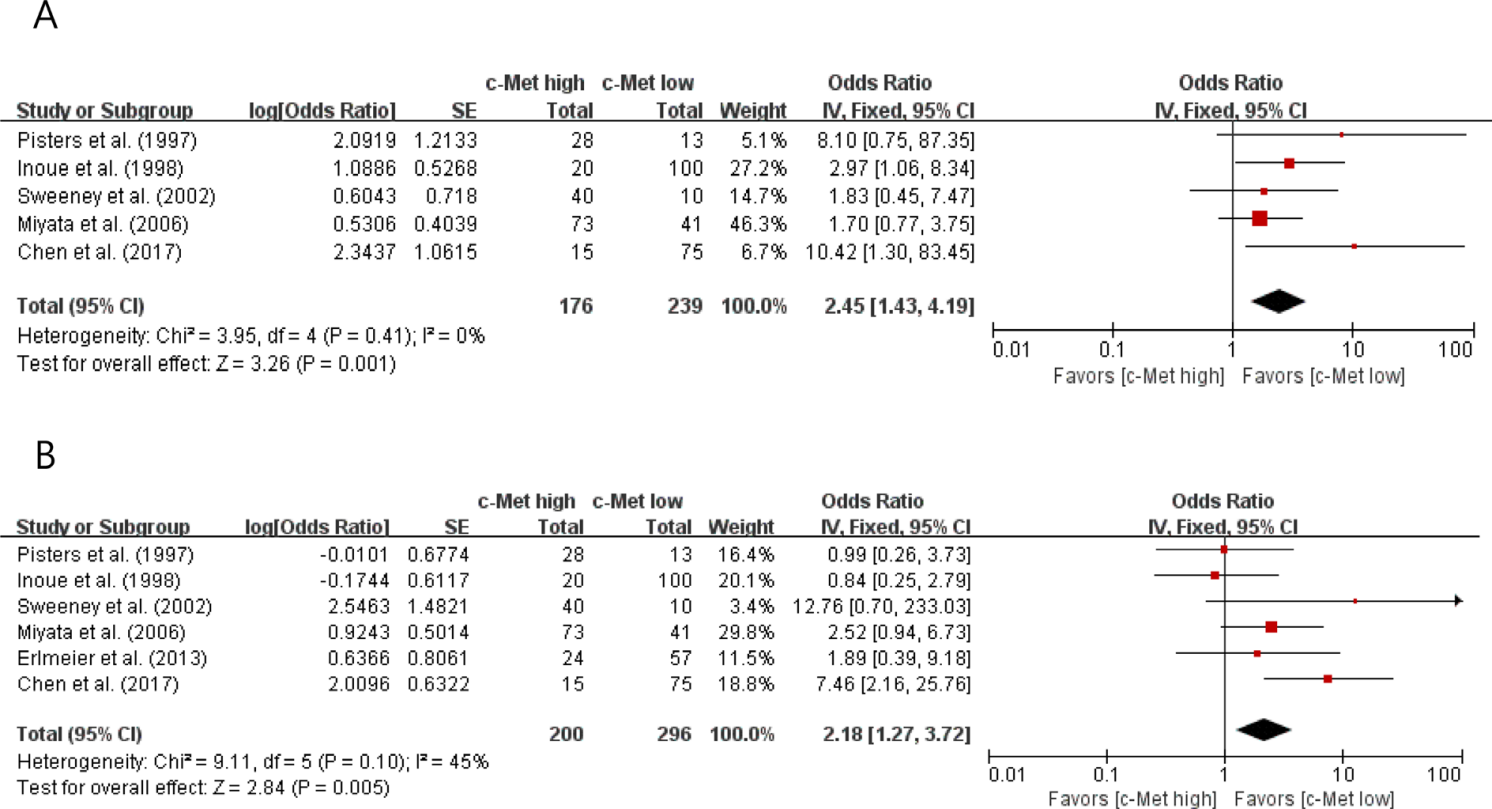
Forest plots of odds ratios for nuclear grade (A) and pT stage (B)

Compared with RCCs with low c-Met expression, tumors with high c-Met expression showed significantly higher nuclear grade (II–IV) (OR = 2.45 [95% CI: 1.43–4.19], *P* = 0.001) (Figure 2A). The fixed-effects model was selected because there was no significant heterogeneity among studies (*X*^2^ = 3.95, *P* = 0.41, *I*^*2*^ = 0%).

In terms of primary tumor stage, there was a positive correlation between c-Met overexpression and higher pT stage (pT3 and pT4) (OR = 2.18 [95% CI: 1.27–3.72], *P* = 0.005) (Figure 2B). The fixed-effects model was used because there was little heterogeneity across studies (*X*^2^ = 9.11, *P* = 0.10, *I*^*2*^ = 45%).

### Impact of high c-Met expression on overall survival

**Figure 3:**
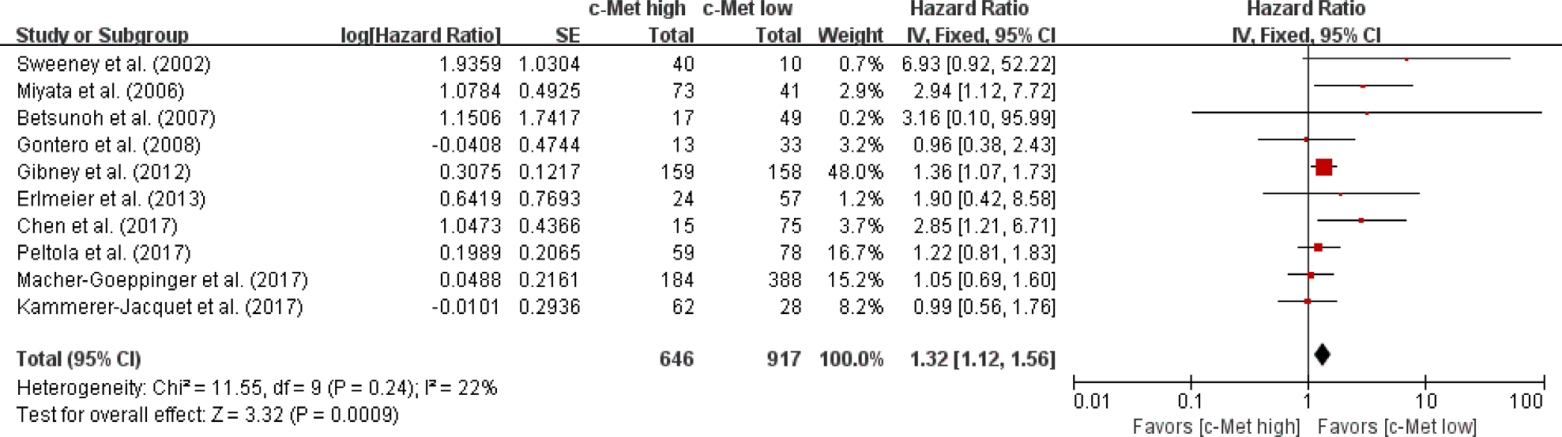
Forest plot of hazard ratios for overall survival

From ten studies [30–39], 1,563 patients were included in the meta-analysis of HRs with 95% CIs for OS. Patients with c-Met-high RCC showed significantly worse OS than those with c-Met-low tumor (HR = 1.32 [95% CI: 1.12–1.56], *P* = 0.0009) (Figure 3). The fixed-effects model was selected because there was no significant heterogeneity across the studies (*X*^2^ = 11.55, *P* = 0.24, *I*^*2*^ = 22%).

**Figure 4:**
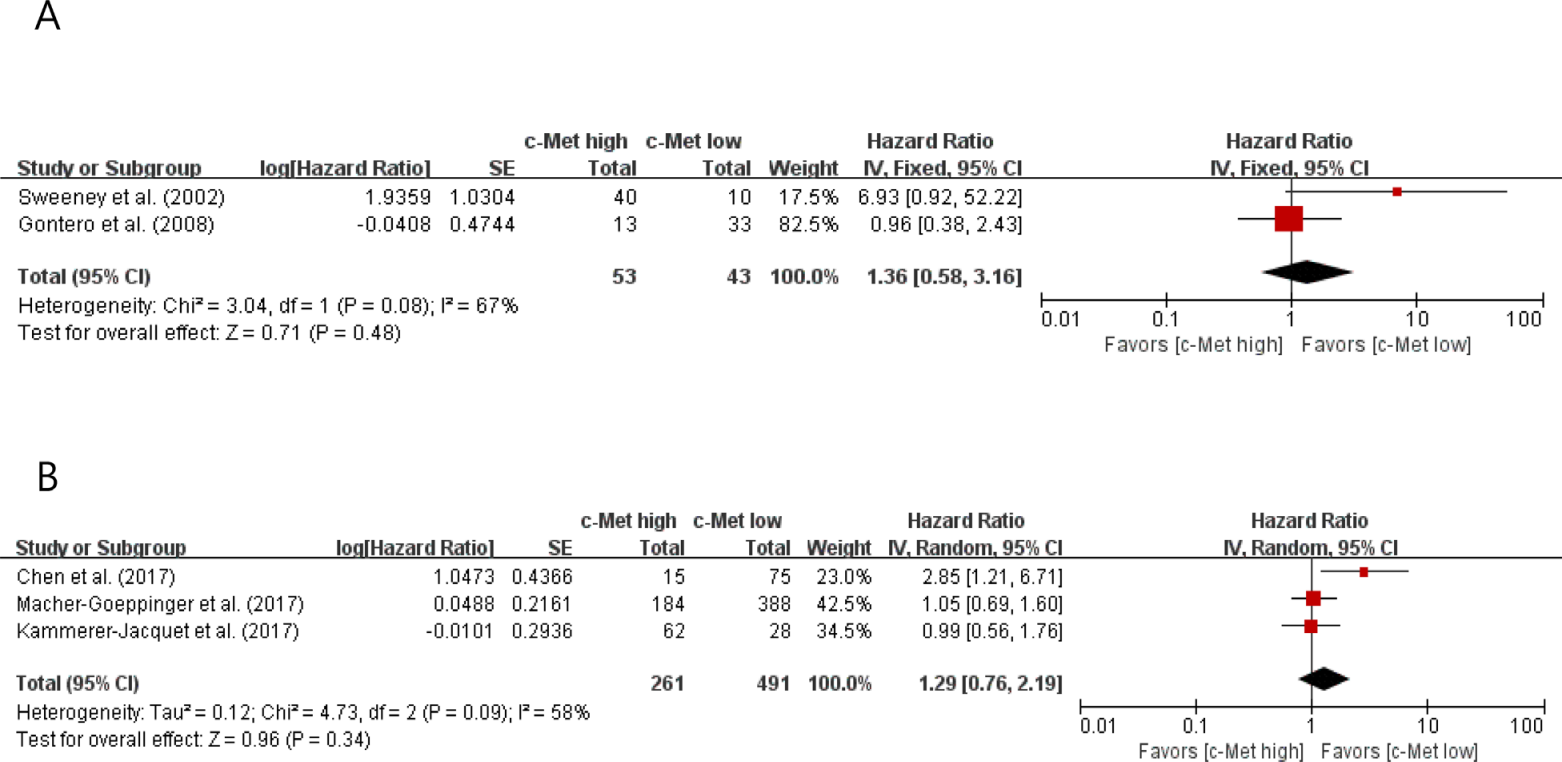
Forest plots of hazard ratios for overall survival in papillary RCC (A) and clear cell RCC (B)

In the subgroup analyses, however, patients with c-Met-high tumor did not show significantly worse OS than those with c-Met-low tumor both in pRCC (HR = 1.36 [95% CI: 0.58–3.16], *P* = 0.48) (Figure 4A) and ccRCC (HR = 1.29 [95% CI: 0.76–2.19], *P* = 0.34) (Figure 4B).

### Publication bias

**Figure 5:**
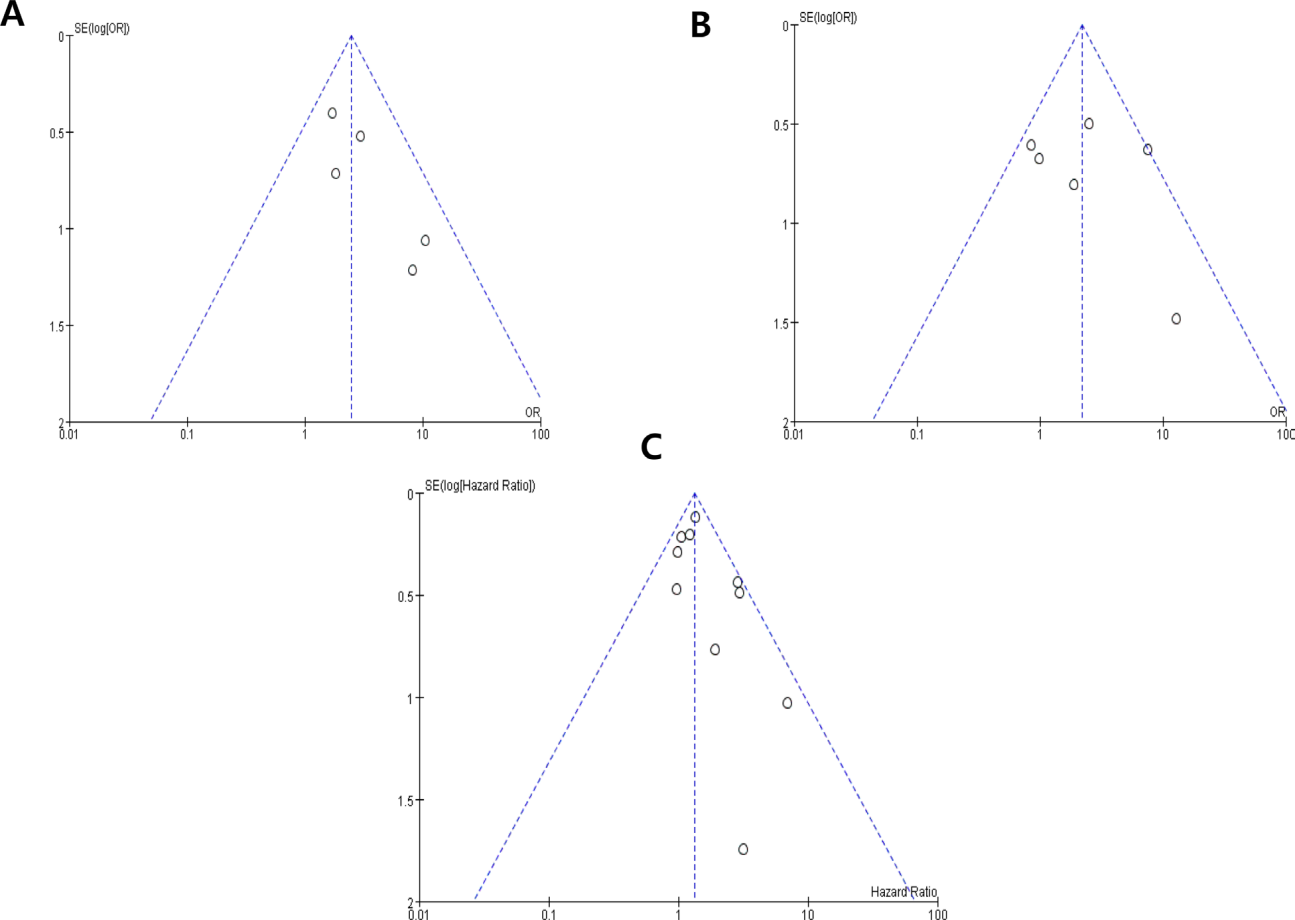
Funnel plots for publication bias regarding nuclear grade (A) pT stage (B) and overall survival (C)

Visual inspection of the funnel plots for nuclear grade, pT stage, and OS showed symmetry, indicating there were no substantial publication biases (Figure 5).

## DISCUSSION

In this meta-analysis, we evaluated the pathologic and prognostic impact of c-Met overexpression in patients with RCC. The results show that high c-Met expression is associated with significantly worse pathological features and prognosis.

*MET* activation has been proven to play a critical role in the pathogenesis and progression of many tumor types [14–16]. Mechanisms of *MET* activation include mutations, amplification, and enhanced transcription [40]. Germline *MET* mutations have been identified in hereditary and sporadic pRCC [41]. c-Met was overexpressed in von Hippel-Lindau (VHL) RCC cells due to the upregulation of hypoxia-inducible factors [42]. In addition, VHL mutation and/or loss of heterozygosity have been associated with c-Met expression in ccRCC [25]. Besides pRCC and ccRCC, *MET* upregulation has also been detected in a rarer subtype, chromophobe RCC [35].

In RCC, many studies have suggested that c-Met expression is associated with significantly inferior clinicopathological features, such as tumor grade [27, 28–31, 36, 38], primary tumor stage [30, 31, 36], lymphatic invasion [27], metastases [30, 35, 38], and worse progression-free survival [37] or OS [31, 34, 36]. However, the pathological or clinical impacts of c-Met expression are not consistent across the studies [33, 38, 39]. In addition, because most studies had a small number of patients and adopted various methods and criteria for c-Met expression status, they could not draw a consensus regarding the clinicopathological roles of c-Met overexpression. With respect to cancer-specific survival, in particular, the prognostic value of c-Met overexpression has been controversial. Gibney *et al.* evaluated c-Met expression as a prognostic marker in 317 patients with RCC and found that high c-Met expression was an independent predictor of survival (multivariate HR = 1.013 [95% CI: 1.002–1.023], *P* = 0.015) [34]. Recently, Macher-Goeppinger *et al.* assessed c-Met expression and *MET* copy number in 572 patients with ccRCC [38]. Patients with high c-Met expression showed significantly worse OS than those with c-Met-low tumor (HR = 1.49 [95% CI: 1.11–2.0], *P* = 0.008) in univariate analysis. In multivariate analysis, however, c-Met overexpression did not remain as an independent prognostic factor (HR = 1.05 [95% CI: 0.69–1.61), *P* = 0.81].

In the current meta-analysis, we included studies comparing the major pathological features (nuclear grade and pT stage) and cancer-specific survival outcome according to the c-Met expression status. Chen *at al.* had also performed the similar meta-analysis regarding clinicopatholigical impacts of c-Met expression in RCC [36]. They included studies with no criteria or low threshold for c-Met expression. In this study, however, we excluded two articles with no criteria for c-Met expression [24, 25] and another two with very low cutoff value (IHC staining in < 10% of tumor cells) for high c-Met expression [26, 27]. Compared with RCCs showing low c-Met expression, tumors with high expression showed significantly higher nuclear grade (OR = 2.45, *P* = 0.001) and pT stage (OR = 2.18, *P* = 0.005). In addition, patients with c-Met-high RCC showed significantly worse OS than those with c-Met-low tumor (HR = 1.32, *P* = 0.0009). Our findings indicate that high c-Met expression represent a potential adverse prognostic marker for patients with RCC. In the subgroup analyses, however, the OS failed to show statistically significant difference between patients with c-Met-high tumor and those with c-Met-low tumor in both pRCC (HR = 1.36, *P* = 0.48) and ccRCC (HR = 1.29, *P* = 0.34). Because the limited number of studies was included in the subgroup analyses, further studies are needed to evaluate the prognostic role of c-Met expression in the subtypes of RCC.

Several meta-analyses in other cancers have also demonstrated that high c-Met expression is an adverse prognostic marker for survival [17–23]. Thus, inhibition of c-Met/HGF pathway may provide an effective therapeutic strategy for cancers with c-Met overexpression [43]. Based on the scientific rationale to target c-Met, various c-Met inhibitors have been investigated in a variety of cancers, including RCC [12, 13, 44–49]. Cabozantinib is an oral inhibitor of tyrosine kinases including MET, VEGFR, and AXL. The randomized phase 3 METEOR trial compared the efficacy and safety of cabozantinib versus the mTOR inhibitor everolimus in patients with advanced RCC who progressed after previous VEGFR tyrosine-kinase inhibitor treatment [49]. Compared with everolimus, cabozantinib significantly prolonged OS (median 21·4 vs. 16·5 months, HR = 0.66 [95% CI: 0·53–0·83], *P* = 0·00026) and progression-free survival (median 7.4 vs. 3.9 months, HR = 0·51 [95% CI: 0·41–0·62], *P* < 0·0001). Based on these results, the U. S. FDA approved cabozantinib for patients with advanced RCC who have received prior anti-angiogenic therapy. In a phase II study with RCC, interestingly, therapeutic response of foretinib (a multi-kinase inhibitor targeting c-Met, VEGF, RON, AXL, and TIE-2 receptors) was closely associated with the germline *MET* mutations [12]. In addition, the efficacy of c-Met-targeting agents has been associated with high c-Met expression in non-small-cell lung cancer and hepatocellular carcinoma [46, 47]. These results suggest that cancers showing high c-Met expression may be good candidates for c-Met inhibitors.

Recently, *MET* amplification/upregulation has been proposed as a mechanism of resistance to anti-angiogenic therapy [46, 47]. Anti-angiogenic therapy induces hypoxia by decreasing blood supply to tumors, which may upregulate c-Met [42]. c-Met activation in turn promotes tumor invasion and metastasis [16]. Peltola *et al.* retrospectively analyzed c-Met expression in 137 patients with metastatic RCC treated with sunitinib and found that high c-Met expression was associated with poor survival [38]. c-Met upregulation by anti-VEGF therapy may explain the reason why patients with high c-Met expression achieved less survival benefit from sunitinib. This finding indicates that c-Met expression may serve as a biomarker to predict who benefit less from anti-angiogenic therapy. Therefore, those patients with c-Met overexpression might benefit from a dual inhibitor [48].

The major limitation for clinical application of c-Met inhibitors is that there is no consensus of the reliable criteria for high c-Met expression. A variety of methods, such as IHC, Western blot, fluorescence *in situ* hybridization, reverse transcription-polymerase chain reaction (RT-PCR), and molecular invasion probe are currently used to assess c-Met status, but there are no standardized criteria for overexpression. There are also differences in the IHC criteria for high c-Met expression. The discrepancies in the clinicopathological impacts of c-Met among studies might be due to the different methods and criteria for high c-Met expression. Therefore, the definition of reliable criteria for c-Met status is essential to verify the prognostic role of c-Met expression and develop c-Met inhibitors in solid tumors.

Our study has several inherent limitations. First, the meta-analysis included a small number of studies. Second, all the studies were retrospectively performed. Third, as we mentioned above, the studies used different IHC methods (antibodies, detection kits, tissue samples-whole slide or tissue microarray, staining sites-cytoplasm or membrane) for assessing c-Met expression. In addition IHC criteria to stratify c-Met status were also various among studies. Fourth, because of limited information in most studies, we could not consider the impact of systemic therapies that would have inevitably affected the OS. Finally articles published only in English were included, which might bias the results.

In conclusion, our results show that c-Met overexpression is significantly associated with poor pathological features and prognosis. These findings indicate that high c-Met expression is a potential adverse prognostic marker for patients with RCC. However, larger studies using standardized methods and criteria are still needed to verify the prognostic roles of c-Met expression in various subtypes of RCC.

## MATERIALS AND METHODS

### Search strategy

We performed this meta-analysis according to the Preferred Reporting Items for Systematic Reviews and Meta-Analyses (PRISMA) guidelines [50]. A systematic computerized search of the electronic databases PubMed and Embase (up to May 2017) was done. The search used the following terms: “c-Met” or “Met,” “hepatocyte growth factor receptor,” and “renal cell carcinoma.” The related articles function in the PubMed was also used to identify all related articles. The titles and abstracts of the retrieved studies were initially scanned to exclude irrelevant papers. Then, the potentially relevant articles were reviewed in full text, further excluding those that did not meet the inclusion criteria of this meta-analysis.

### Inclusion criteria

Eligible studies should meet the following inclusion criteria: (i) patients had a pathological diagnosis of RCC; (ii) pathological features (nuclear grade and/or pT stage) or OS were analyzed according to c-Met expression status; (iii) ORs with 95% CIs for pathological features or HR with 95% CI for OS were provided or could be estimated from the data provided; (iv) articles were published in English.

Articles with no criteria for c-Met expression status were excluded. We also excluded articles with very low cutoff value (IHC staining in < 10% of tumor cells) for high c-Met expression.

### Data extraction

The required data were collected independently by two investigators (BJK and JHK). If these two authors did not agree, the other investigator (HSK) was consulted to resolve the discrepancies.

The following data were recorded from all eligible studies: the first author’s name, publication year, country, histology, nuclear grade, pT stage, methods to test c-Met expression, antibody and detection kit for IHC, number of patients, treatment, cutoff values adopted to dichotomize c-Met expression as ‘high’ or ‘low’, and HR with 95% CI for OS and OR with 95% CI for pathological features.

### Statistical analysis

Statistical values were obtained directly from the original articles. When OR or HR with 95% CI were not provided, the Engauge Digitizer (version 9.1) was used to estimate the needed data from the results and Kaplan-Meier curves. The strength of the association between c-Met overexpression and nuclear grade or pT stage was shown as ORs with their 95% CIs. The effect size of OS was pooled through HR with its 95% CI. The heterogeneity across studies was tested by the *Q* statistic and the *I*^2^ inconsistency test. The fixed-effects model (Mantel–Haenszel method) was selected for pooling homogeneous outcomes when *P* ≥ 0.1 and *I*^2^ *≤* 50%, whereas the random-effects model (DerSimonian–Laird method) was applied for pooling heterogeneous outcomes when *P* <  0.1 and *I*^2^ > 50%. The RevMan (version 5.2) was used to combine data and report outcomes. All reported *P*-values were two-sided and *P* < 0.05 was considered statistically significant. Publication bias was assessed graphically by the funnel plot method [51].

## References

[1] Ferlay J, Soerjomataram I, Dikshit R, Eser S, Mathers C, Rebelo M, Parkin DM, Forman D, Bray F (2015). Cancer incidence and mortality worldwide: sources, methods and major patterns in GLOBOCAN 2012. Int J Cancer.

[2] Won YJ, Oh CM, Kong HJ, Lee DH, Lee KH (2017). Community of Population-Based Regional Cancer Registries. Cancer statistics in Korea: incidence, mortality, survival, and prevalence in 2014. Cancer Res Treat.

[3] Siegel RL, Miller KD, Jemal A (2016). Cancer statistics, 2016. CA Cancer J Clin.

[4] Negrier S, Escudier B, Lasset C, Douillard JY, Savary J, Chevreau C, Ravaud A, Mercatello A, Peny J, Mousseau M, Philip T, Tursz T (1998). Recombinant human interleukin-2, recombinant human interferon alfa-2a, or both in metastatic renal-cell carcinoma. Groupe Français d’Immunothérapie. N Engl J Med.

[5] Pantuck AJ, Belldegrun AS, Figlin RA (2001). Nephrectomy and interleukin-2 for metastatic renal-cell carcinoma. N Engl J Med.

[6] Escudier B, Eisen T, Stadler WM, Szczylik C, Oudard S, Siebels M, Negrier S, Chevreau C, Solska E, Desai AA, Rolland F, Demkow T, Hutson TE (2007). Sorafenib in advanced clear-cell renal-cell carcinoma. N Engl J Med.

[7] Motzer RJ, Escudier B, Oudard S, Hutson TE, Porta C, Bracarda S, Grünwald V, Thompson JA, Figlin RA, Hollaender N, Urbanowitz G, Berg WJ, Kay A (2008). Efficacy of everolimus in advanced renal cell carcinoma: a double-blind, randomised, placebo-controlled phase III trial. Lancet.

[8] Rini BI, Halabi S, Rosenberg JE, Stadler WM, Vaena DA, Ou SS, Archer L, Atkins JN, Picus J, Czaykowski P, Dutcher J, Small EJ (2010). Bevacizumab plus interferon alfa compared with interferon alfa monotherapy in patients with metastatic renal cell carcinoma: CALGB 90206. J Clin Oncol.

[9] Sternberg CN, Davis ID, Mardiak J, Szczylik C, Lee E, Wagstaff J, Barrios CH, Salman P, Gladkov OA, Kavina A, Zarbá JJ, Chen M, McCann L (2010). Pazopanib in locally advanced or metastatic renal cell carcinoma: results of a randomized phase III trial. J Clin Oncol.

[10] Hudes G, Carducci M, Tomczak P, Dutcher J, Figlin R, Kapoor A, Staroslawska E, Sosman J, McDermott D, Bodrogi I, Kovacevic Z, Lesovoy V, Schmidt-Wolf IG (2007). Temsirolimus, interferon alfa, or both for advanced renal-cell carcinoma. N Engl J Med.

[11] Blumenschein GR, Mills GB, Gonzalez-Angulo AM (2012). Targeting the hepatocyte growth factor-cMET axis in cancer therapy. J Clin Oncol.

[12] Choueiri TK, Vaishampayan U, Rosenberg JE, Logan TF, Harzstark AL, Bukowski RM, Rini BI, Srinivas S, Stein MN, Adams LM, Ottesen LH, Laubscher KH, Sherman L (2013). Phase II and biomarker study of the dual MET/VEGFR2 inhibitor foretinib in patients with papillary renal cell carcinoma. J Clin Oncol.

[13] Choueiri TK, Halabi S, Sanford BL, Hahn O, Michaelson MD, Walsh MK, Feldman DR, Olencki T, Picus J, Small EJ, Dakhil S, George DJ, Morris MJ (2017). Cabozantinib versus sunitinib as initial targeted therapy for patients with metastatic renal cell carcinoma of poor or intermediate risk: The Alliance A031203 CABOSUN Trial. J Clin Oncol.

[14] Furge KA, Zhang YW (2000). Vande Woude GF. Met receptor tyrosine kinase: enhanced signaling through adapter proteins. Oncogene.

[15] Rosário M, Birchmeier W (2003). How to make tubes: signaling by the Met receptor tyrosine kinase. Trends Cell Biol.

[16] Zhang YW, Su Y, Volpert OV, VandeWoude GF (2003). Hepatocyte growth factor/scatter factor mediates angiogenesis through positive VEGF and negative thrombospondin 1 regulation. Proc Natl Acad Sci USA.

[17] Yan S, Jiao X, Zou H, Li K (2015). Prognostic significance of c-Met in breast cancer: a meta-analysis of 6010 cases. Diagn Pathol.

[18] Pyo JS, Kang G, Cho WJ, Choi SB (2016). Clinicopathological significance and concordance analysis of c-MET immunohistochemistry in non-small cell lung cancers: A meta-analysis. Pathol Res Pract.

[19] Yu S, Yu Y, Zhao N, Cui J, Li W, Liu T (2013). C-Met as a prognostic marker in gastric cancer: a systematic review and meta-analysis. PLoS One.

[20] Liu Y, Yu XF, Zou J, Luo ZH (2015). Prognostic value of c-Met in colorectal cancer: a meta-analysis. World J Gastroenterol.

[21] Peng J, Qi S, Wang P, Li W, Liu C, Li F (2016). Diagnosis and prognostic significance of c-Met in cervical cancer: a meta-analysis. Dis Markers.

[22] Kim JH, Kim HS, Kim BJ, Lee J, Jang HJ (2017). Prognostic value of c-Met overexpression in pancreatic adenocarcinoma: a meta-analysis. Oncotarget.

[23] Kim JH, Kim HS, Kim BJ, Jang HJ, Lee J (2017). Prognostic value of c-Met overexpression in hepatocellular carcinoma: a meta-analysis and review. Oncotarget.

[24] Mukai S, Yorita K, Kawagoe Y, Katayama Y, Nakahara K, Kamibeppu T, Sugie S, Tukino H, Kamoto T, Kataoka H (2015). Matriptase and MET are prominently expressed at the site of bone metastasis in renal cell carcinoma: immunohistochemical analysis. Hum Cell.

[25] Oh RR, Park JY, Lee JH, Shin MS, Kim HS, Lee SK, Kim YS, Lee SH, Lee SN, Yang YM, Yoo NJ, Lee JY, Park WS (2002). Expression of HGF/SF and Met protein is associated with genetic alterations of VHL gene in primary renalcell carcinomas. APMIS.

[26] Natali PG, Prat M, Nicotra MR, Bigotti A, Olivero M, Comoglio PM, Di Renzo MF (1996). Overexpression of the met/HGF receptor in renalcell carcinomas. Int J Cancer.

[27] Choi JS, Kim MK, Seo JW, Choi YL, Kim DH, Chun YK, Ko YH (2006). MET expression in sporadic renal cell carcinomas. J Korean Med Sci.

[28] Pisters LL, el-Naggar AK, Luo W, Malpica A, Lin SH (1997). C-met proto-oncogene expression in benign and malignant human renal tissues. J Urol.

[29] Inoue K, Karashima T, Chikazawa M, Iiyama T, Yoshikawa C, Furihata M, Ohtsuki Y, Shuin T (1998). Overexpression of c-met proto-oncogene associated with chromophilic renal cell carcinoma with papillary growth. Virchows Arch.

[30] Sweeney P, El-Naggar AK, Lin SH, Pisters LL (2002). Biological significance of c-met over expression in papillary renal cell carcinoma. J Urol.

[31] Miyata Y, Kanetake H, Kanda S (2006). Presence of phosphorylated hepatocyte growth factor receptor/c-Met is associated with tumor progression and survival in patients with conventional renal cell carcinoma. Clin Cancer Res.

[32] Betsunoh H, Mukai S, Akiyama Y, Fukushima T, Minamiguchi N, Hasui Y, Osada Y, Kataoka H (2007). Clinical relevance of hepsin and hepatocyte growth factor activator inhibitor type 2 expression in renal cell carcinoma. Cancer Sci.

[33] Gontero P, Ceratti G, Guglielmetti S, Andorno A, Terrone C, Bonvini D, Faggiano F, Tizzani A, Frea B, Valente G (2008). Prognostic factors in a prospective series of papillary renal cell carcinoma. BJU Int.

[34] Gibney GT, Aziz SA, Camp RL, Conrad P, Schwartz BE, Chen CR, Kelly WK, Kluger HM (2013). c-Met is a prognostic marker and potential therapeutic target in clear cell renal cell carcinoma. Ann Oncol.

[35] Erlmeier F, Ivanyi P, Hartmann A, Autenrieth M, Wiedemann M, Weichert W, Steffens S (2017). c-Met in chromophobe renal cell carcinoma. Med Oncol.

[36] Chen S, Zhu Y, Cui J, Wang Y, Xia Y, Song J, Cheng S, Zhou C, Zhang D, Zhang B, Shi B The role of c-Met in prognosis and clinicopathology of renal cell carcinoma: Results from a single-centre study and systematic review. Urol Oncol.

[37] Peltola KJ, Penttilä P, Rautiola J, Joensuu H, Hänninen E, Ristimäki A, Bono P Correlation of c-Metexpression and outcome in patients with renal cell carcinomatreated with sunitinib. Clin Genitourin Cancer.

[38] Macher-Goeppinger S, Keith M, Endris V, Penzel R, Tagscherer KE, Pahernik S, Hohenfellner M, Gardner H, Grüllich C, Schirmacher P, Roth W (2017). MET expression and copy number status in clear-cellrenal cell carcinoma: prognostic value and potential predictive marker. Oncotarget.

[39] Kammerer-Jacquet SF, Medane S, Bensalah K, Bernhard JC, Yacoub M, Dupuis F, Ravaud A, Verhoest G, Mathieu R, Peyronnet B, Brunot A, Laguerre B, Lespagnol A (2017). Correlation of c-MET Expression with PD-L1 Expression in Metastatic Clear Cell Renal Cell Carcinoma Treated by Sunitinib First-Line Therapy. Target Oncol.

[40] Danilkovitch-Miagkova A, Zbar B (2002). Dysregulation of Met receptor tyrosine kinase activity in invasive tumors. J Clin Invest.

[41] Schmidt L, Duh FM, Chen F, Kishida T, Glenn G, Choyke P, Scherer SW, Zhuang Z, Lubensky I, Dean M, Allikmets R, Chidambaram A, Bergerheim UR (1997). Germline and somatic mutations in the tyrosine kinase domain of the MET proto-oncogene in papillary renal carcinomas. Nat Genet.

[42] Nakaigawa N, Yao M, Baba M, Kato S, Kishida T, Hattori K, Nagashima Y, Kubota Y (2006). Inactivation of von Hippel-Lindau gene induces constitutive phosphorylation of MET protein in clear cell renal carcinoma. Cancer Res.

[43] Peters S, Adjei AA (2012). MET: a promising anticancer therapeutic target. Nat Rev Clin Oncol.

[44] Shojaei F, Lee JH, Simmons BH, Wong A, Esparza CO, Plumlee PA, Feng J, Stewart AE, Hu-Lowe DD, Christensen JG (2010). HGF/c-Met acts as an alternative angiogenic pathway in sunitinib-resistant tumors. Cancer Res.

[45] Jahangiri A, De Lay M, Miller LM, Carbonell WS, Hu YL, Lu K, Tom MW, Paquette J, Tokuyasu TA, Tsao S, Marshall R, Perry A, Bjorgan KM (2013). Gene expression profile identifies tyrosine kinase c-Met as a targetable mediator of antiangiogenic therapy resistance. Clin Cancer Res.

[46] Spigel DR, Ervin TJ, Ramlau RA, Daniel DB, Goldschmidt JH, Blumenschein GR, Krzakowski MJ, Robinet G, Godbert B, Barlesi F, Govindan R, Patel T, Orlov SV (2013). Randomized phase II trial of onartuzumab in combination with erlotinib in patients with advanced non-small-cell lung cancer. J Clin Oncol.

[47] Santoro A, Rimassa L, Borbath I, Daniele B, Salvagni S, Van Laethem JL, Van Vlierberghe H, Trojan J, Kolligs FT, Weiss A, Miles S, Gasbarrini A, Lencioni M (2013). Tivantinib for second-line treatment of advanced hepatocellular carcinoma: a randomised, placebo-controlled phase 2 study. Lancet Oncol.

[48] Xiang Q, Chen W, Ren M, Wang J, Zhang H, Deng DY, Zhang L, Shang C, Chen Y (2014). Cabozantinib suppresses tumor growth and metastasis in hepatocellular carcinoma by a dual blockade of VEGFR2 and MET. Clin Cancer Res.

[49] Choueiri TK, Escudier B, Powles T, Tannir NM, Mainwaring PN, Rini BI, Hammers HJ, Donskov F, Roth BJ, Peltola K, Lee JL, Heng DY, Schmidinger M (2016). Cabozantinib versus everolimus in advanced renal cell carcinoma (METEOR): final results from a randomised, open-label, phase 3 trial. Lancet Oncol.

[50] Panic N, Leoncini E, de Belvis G, Ricciardi W, Boccia S (2013). Evaluation of the endorsement of the preferred reporting items for systematic reviews and meta-analysis (PRISMA) statement on the quality of published systematic review and meta-analyses. PLoS One.

[51] Sterne JA, Sutton AJ, Ioannidis JP, Terrin N, Jones DR, Lau J, Carpenter J, Rücker G, Harbord RM, Schmid CH, Tetzlaff J, Deeks JJ, Peters J (2011). Recommendations for examining and interpreting funnel plot asymmetry in meta-analyses of randomised controlled trials. BMJ.

